# Nitric Oxide and TNFα Are Critical Regulators of Reversible Lymph Node Vascular Remodeling and Adaptive Immune Response

**DOI:** 10.1371/journal.pone.0060741

**Published:** 2013-04-03

**Authors:** Stephanie L. Sellers, Akiko Iwasaki, Geoffrey W. Payne

**Affiliations:** 1 Interdisciplinary Studies, University of Northern BC, Prince George, British Columbia, Canada; 2 Immunobiology, Yale School of Medicine, New Haven, Connecticut, United States of America; 3 Northern Medical Program, University of Northern BC, Prince George, British Columbia, Canada; Idaho State University, United States Of America

## Abstract

Lymph node (LN) vascular growth, at the level of the main arteriole, was recently characterized for the first time during infection. Arteriole diameter was shown to increase for at least seven days and to occur via a CD4^+^ T cell dependent mechanism, with vascular expansion playing a critical role in regulating induction of adaptive immune response. Here, using intravital microscopy of the inguinal LN during herpes simplex type II (HSV-2) infection, the data provides the first studies that demonstrate arteriole expansion during infection is a reversible vascular event that occurs via eutrophic outward remodeling. Furthermore, using genetic ablation models, and pharmacological blockade, we reveal arteriole remodeling and LN hypertrophy to be dependent upon both endothelial nitric oxide synthase (eNOS) and TNFα expression. Additionally, we reveal transient changes in nitric oxide (NO) levels to be a notable feature of response to viral infection and LN vascular remodeling and provide evidence that mast cells are the critical source of TNFα required to drive arteriole remodeling. Overall, this study is the first to fully characterize LN arteriole vascular changes throughout the course of infection. It effectively reveals a novel role for NO and TNFα in LN cellularity and changes in LN vascularity, which represent key advances in understanding LN vascular physiology and adaptive immune response.

## Introduction

During infection lymph nodes (LNs) serve as an interface between the innate and adaptive immune systems. As secondary lymphoid organs, the chief function of LNs is in collecting antigen and antigen presenting cells from the periphery that subsequently present antigen to naïve lymphocytes that traffic to the LNs. An intricate vascular network facilitates the trafficking of naïve lymphocytes to and from LNs, with the main feed arteriole being the upstream supplier of blood. This arteriole branches into a capillary network and then in to specialized post-capillary high endothelial venules (HEVs). HEVs directly feed LNs and are specialized to facilitate movement of cells into the LN and are therefore critical to successful lymphocyte migration into the LN and immune response [1]–[3]. As such, extensive research regarding signaling molecules and chemokine expression patterns in HEVs as well as studies of general studies of LN blood flow and vascular expansion in HEVs and lymphatic drainage have been reported. However, until recently the specific role of blood vessels upstream of HEVs in regulating blood flow to LNs and HEVs during immunization or their role in LN function during immune response remained unexplored.

The LN feed arteriole is the upstream blood supply to HEVs infiltrating the LN [1]. The first study to focus on the LN feed arteriole during infection identified it as a key regulatory of immune response, by demonstrating that three days post-infection with HSV-2 there is significant remodeling of the feed arteriole to a larger diameter [4]. This increase in maximal vessel diameter was noted to be 50% and was found to facilitate increased blood flow and supply of naïve T cells to the LN, and thereby increased the rate of screening for rare cognate lymphocytes from 3×10^6^ cells/day, which has been noted in a naïve mouse, to 14×10^6^ cells/day [4], [5]. Increased arteriole diameter was also noted to correspond with cellular accumulation and HEV proliferation [4].

After screening for naïve lymphocytes, LNs are the site of induction of antigen specific cytotoxic T lymphocyte (CTL) responses that are critical to fighting intracellular pathogens. Within the LN, CD4^+^ T cells aid CTL responses in multiple ways including licensing of dendritic cells (DCs) for efficient antigen presentation and recruitment of naïve CD8^+^ T cell [6]–[8]. Recently, our group identified vascular remodeling of the LN feed arteriole to play a critical role in the aspect of immune response by demonstrating a new form of CD4-help for CTL responses in the form of enabling remodeling of the primary LN feed arteriole [9]. The LN arteriole diameter was shown to increase in diameter beginning as early as one day following infection and continuing for at least seven days. Furthermore, arteriole remodeling was dependent upon the presence of CD4^+^ T cells, with arteriole remodeling facilitating CD4^+^ T cell entry into the LN, wherein CD4^+^ T cells interact with DCs through CD40, which is a critical factor in facilitating naïve polyclonal CD8^+^ T cells entry into the LN and governs the magnitude of CTL response to infectious agent [9].

While collectively, previous study of the upstream blood supply to LNs during infection has identified arteriole remodeling to facilitate naïve T cell entry into the LN and CD4^+^ T cell driven vascular growth of the LN feed arteriole as a mediator of adaptive response pathogens, further study of the LN arteriole is clearly needed. Focus on the LN arteriole would contribute significantly to understanding the physiology of LNs and arterioles as well as the function of LN arteriole remodeling during infection and address questions regarding the full extent of remodeling through the complete time course of infection, nature of remodeling, vascular physiology of LN arteriole, and further elucidating the mechanism driving arteriole remodeling and therefore significantly contributing to induction of adaptive immune response. Here, we provide such a contribution by demonstrating changes in arteriole diameter during infection to be a result of outward eutrophic remodeling that is completely reversible. Furthermore, we show in arteriole remodeling and LN hypertrophy that eNOS, TNF**α** and mast cells to be contributing factors to the magnitude of remodeling and the LN arteriole to facilitate transient changes in NO levels through the course of infection.

## Results

### The lymph node feed arteriole exhibits reversible outward eutrophic remodeling

**Figure 1:**
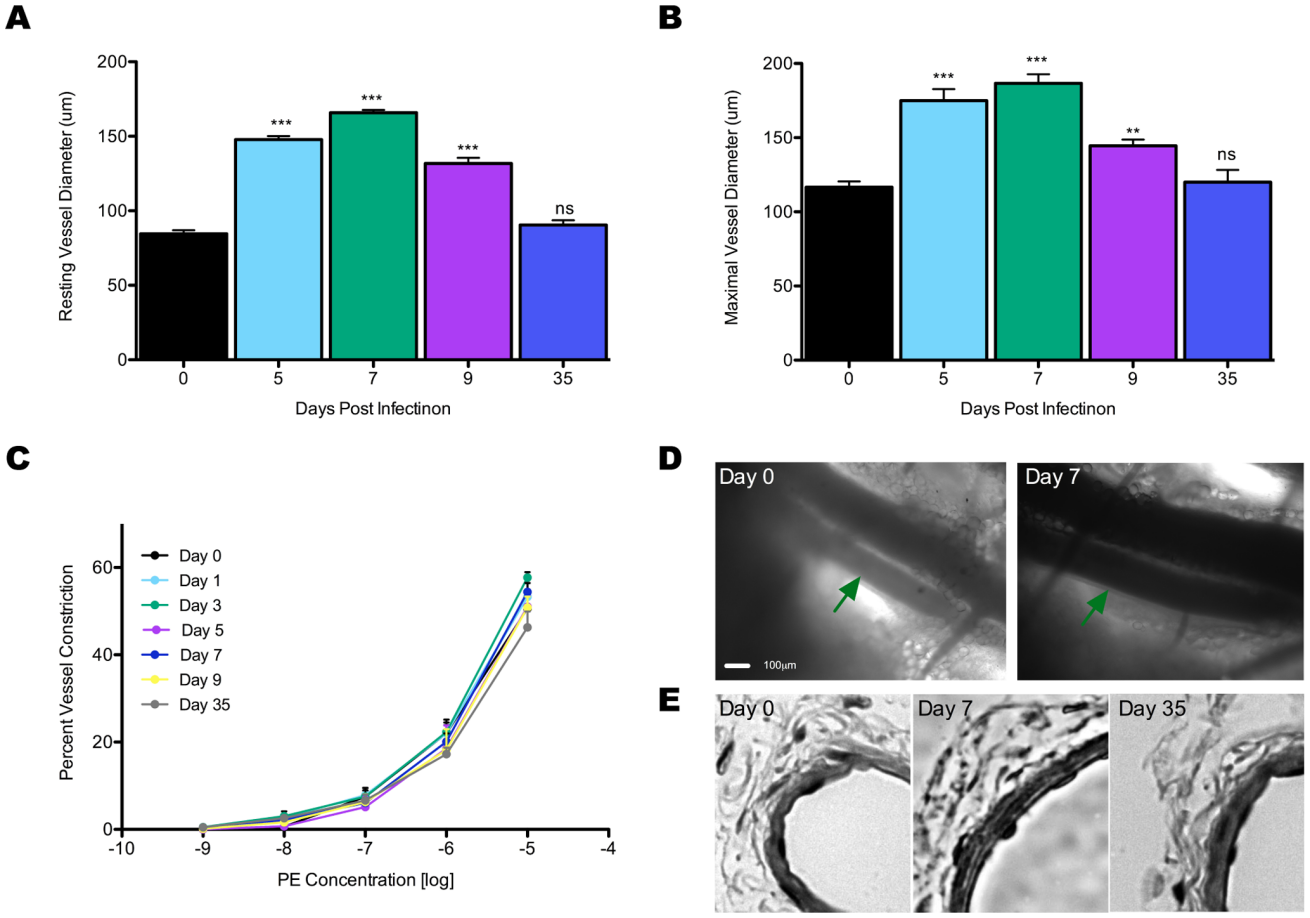
Resting arteriole diameter at days zero, five, seven, nine, and thirty-five (5 weeks) after i.vag. infection with TK^-^HSV-2. Resting vessel diameters were recorded after at least sixty minutes of equilibration with superfused physiological saline solution where vessel diameter, in which arterioles had consistent diameters for at least the last thirty minutes of equilibration (A). Maximal arteriole diameter (+SNP) at days zero, five, seven, nine, and thirty-five (5 weeks) Data represents mean diameter (A,B) ± SEM wherein n = 11 5, 5, 4, and 4 animals for days 0, 5, 7, 9, and 35 respectively (B). Percent vessel constriction with respect to resting vessel diameter of LN arteriole of wild-type at day zero, one, three, five, seven, nine, and thirty-five p.i. in response to PE applied in fold increases from 10^−9^ to 10^−5^M. Data represents percent constriction (A,C) ± SEM were n = 6 for day zero and n = 5for days one, three, five, seven, nine, and thirty-five (C). Representative images of maximal diameter arterioles following infection at days zero and seven after infection In each image the arteriole is indicated by green arrows (D). Representative images of isolated LN arteriole cross-sections H&E stained (E). *p<0.05, **p<0.01, and ***p<0.001.

Original reports of LN arteriole remodeling noted an increase in diameter of approximately 50% three days following infection [4], with subsequent study demonstrating significant increase in resting and maximal arteriole diameter as early as day one and increasing until at least day seven following infection with Tk^-^HSV-2 [9]. However, studies have yet to examine the nature of arteriole remodeling and if the arteriole reverts to its pre-infection size and morphology. To address this, modified bright-field intravital microscopy [10] was used to examine the inguinal LN feed arteriole of C57 BL/6 mice at multiple days after intra-vaginal (i.vag.) infection with Tk^-^HSV-2, which showed increased resting and maximal arteriole diameter peaked at day seven; arteriole diameter began to decrease back towards pre-infection size by day nine and returned to baseline day zero diameters by five weeks following infection (Figure 1A,B). This demonstrates, for the first time, that arteriole remodeling is a dynamic, reversible process that mirrors the course of induction of adaptive immune response.

At all days post immunization, there is a notable difference in resting and maximal vessel diameters (Figures 1A, B), which demonstrates the ability of the vasculature to respond to an applied vasodilators and speaks to the success of the preparation and endothelial function throughout infection. However, we also wanted to consider the preparation integrity in terms of smooth muscle function. To do so, a dose response curve to phenylephrine (PE) in physiological saline solution superfused over the preparation was conducted. Uninfected day zero wild-type controls showed dose-dependent vasocontriction; less than one percent constriction was seen at the lowest concentration of 10^−9^M, but increased significantly, in a dose-dependent manner, and peaked between 50–60% constriction with application of 10^−5^M PE (Figure 1C). Next, to investigate potential changes in smooth muscle response during infection, PE response curves were conducted at days one, three, five, seven, nine, and thirty-five p.i.. Infected mice at all days showed no significant difference in percent vessel constriction compared to uninfected day zero controls at any concentration of PE (Figure 1C). Together, the dose-dependent response to PE and the consistency of the constriction throughout the course of infection demonstrate that smooth muscle function is not impacted during the arteriole remodeling process.

Although response to endothelium dependent vasodilator and smooth muscle dependent vasoconstrictor demonstrate arteriole functionality throughout infection, this does not preclude changes to the vessel wall in terms of structural adaptation. We questioned the nature of the remodeling as definitively determining if arteriole remodeling was outward or inward, and if arteriole wall thickness was affected, is a key first step in determining the mechanism driving LN arteriole expansion. Given that the blood columns and vessel walls were visible during imaging and the blood columns appeared to be much larger following infection without apparent change in vessel wall thickness (Figure 1D), we hypothesized that the arteriole expanded by outward remodeling while maintaining arteriole wall thickness. To test this, we fixed and isolated sections of arteriole *in vivo* at multiple time points following infection. Sections from days zero, seven, nine, and thirty-five were subsequently H&E stained (Figure 1E). All stained sections showed similar wall thickness throughout the course of infection, and indicate an outward eutrophic remodeling event [11]–[13].

### Lymph node arteriole remodeling and hypertrophy is eNOS Dependent

**Figure 2:**
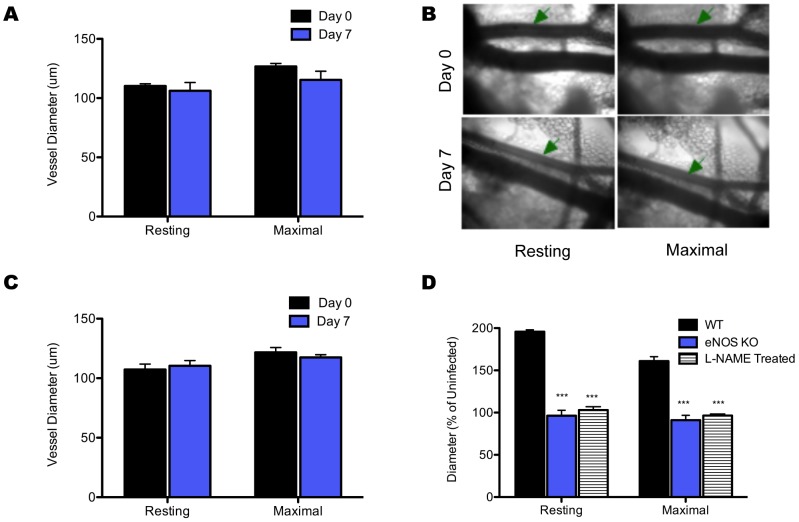
Resting and maximal (+Nifedapine) arteriole diameter at days zero and seven after i.vag. infection in eNOS^-/-^ mice with TK^-^HSV-2 (A). Representative images of resting and maximal diameter eNOS^-/-^ arterioles at days zero and seven after infection where arterioles are indicated by green arrows (B). Resting and maximal (+SNP) arteriole diameter at days zero and seven after i.vag. infection with Tk^-^HSV-2 in mice treated systemically with L-NAME (C). Resting and maximal vessel diameters (% of uninfected) of WT, eNOS^-/-^ and L-NAME treated mice. For eNOS^-/-^ n = 4 and 5 respectively for days zero and seven vessel diameters, and n = 4 for all PE data points (D). For L-NAME treatment, n = 4 for all data points. For WT n = 5 at day seven p.i.. In all cases, one arteriole was studied per animal. *p<0.05, **p<0.01, and ***p<0.001.

Beyond understanding the nature and kinetics of arteriole remodeling during infection, and given that the process of arteriole remodeling drives the magnitude of CTL responses [9], it is also imperative to further elucidate the mechanism of remodeling. Previous reports indicate the presence of CD4^+^ T cells interacting with DCs within the LN were critical for arteriole remodeling and that DCs are responsible for regulating VEGF levels within the LN and facilitating generalized vascular growth via endothelial cell proliferation [9], [14]. Therefore, based on the established importance of eNOS in regulating factors in LN vascular change such as cell migration, vascular growth, endothelial cell proliferation, and modulation of VEGF [15]–[18] in other contexts, we decided to investigate the role of eNOS in arteriole remodeling. Genetic ablation of models lacking expression of eNOS showed an inability to remodel LN arterioles in response to infection (Figure 2A, B). Seven days following infection, the point at which maximal remodeling was seen in wild-type mice, eNOS^-/-^ showed not increase in resting or maximal diameter. To confirm ablation results and address any possibility that lack of remodeling was an artifact of genetic manipulation, we looked at LN arteriole remodeling capacity of wild-type mice treated systemically with NOS inhibitor L-NAME (Figure 2C). In accordance with eNOS^-/-^, pharmacological inhibition of NOS resulted in no significant change in arteriole diameter during infection. This clearly demonstrates the requirement for eNOS expression in facilitating LN arteriole remodeling and is underscored when comparing the percent increase in resting and maximal arteriole diameter relative to day zero diameters in WT, eNOS^-/-^, and L-NAME treated mice at day seven p.i., (Figure 2D) which shows significantly blunted response when eNOS is ablated or NOS function is blocked.

**Figure 3:**
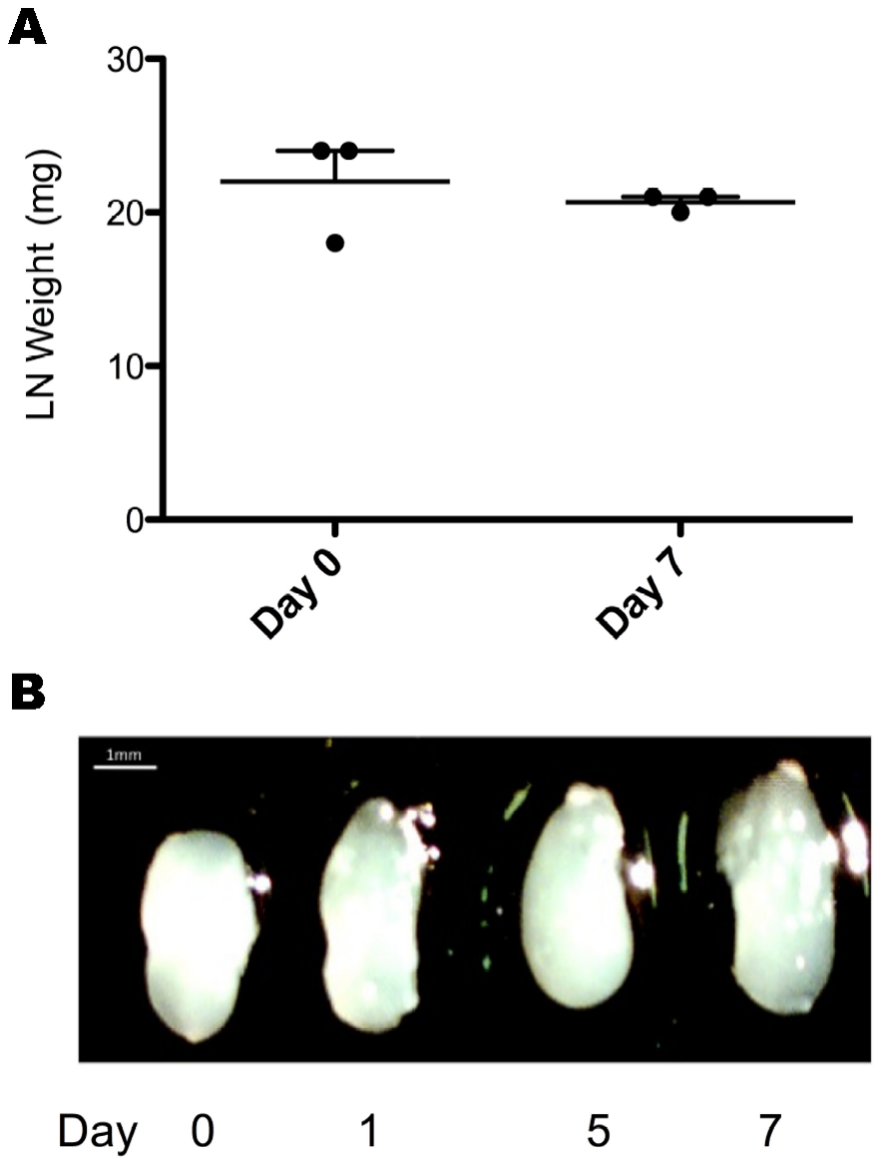
Representative images of isolated eNOS^-/-^ lymph nodes following infection at days zero, one, five, and seven after infection (A). Lymph node weights of isolated eNOS^-/-^ lymph nodes following infection at days zero and seven (B).

Lack of LN arteriole remodeling, as a result of CD4^+^ T cell deficiency, was shown to result in decreased LN hypertrophy due to decreased cellular trafficking [4]. To examine whether eNOS deficiency had the same result, we examined lymph node size and weight following infection. In contrast to wild-type LNs following i.vag. infection with Tk-HSV-2 [9], LNs from eNOS^-/-^ mice showed no increase in gross size throughout the course of infection (Figure 3A). Furthermore, weight of isolated eNOS^-/-^ LNs were similar at days zero and seven following infection (Figure 3B), and demonstrates that eNOS is required for LN arteriole remodeling and subsequently LN hypertrophy.

### Nitric Oxide Levels are Variable During Infection and CD4 Independent

**Figure 4:**
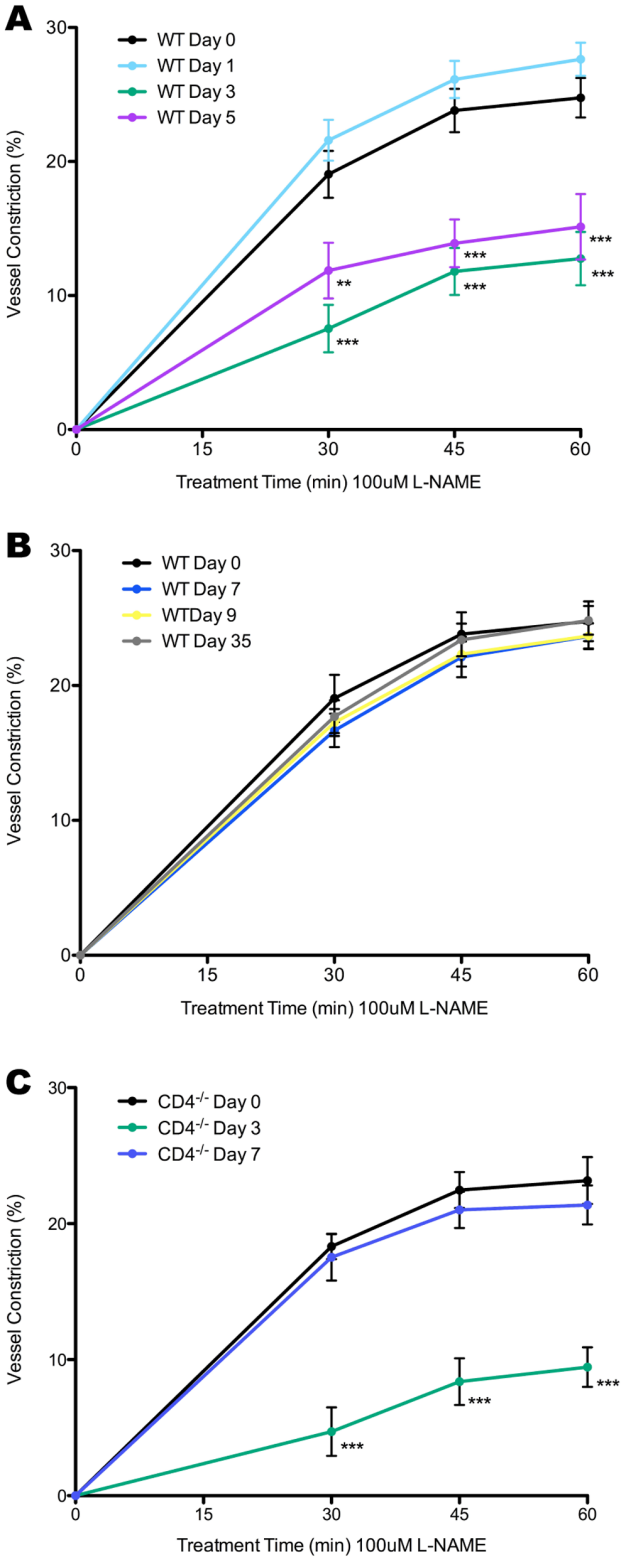
Percentage of vessel constriction in response to perfusion of 100μM L-NAME solution over the vessel preparation. Zero, one, three, and five days p.i. in C57 BL/6 mice (**A)**. Zero, seven, nine, and thirty-five days p.i. in C57 BL/6 mice (**B)**. Zero, three, and seven days p.i. in CD4^-/-^ mice (**C**). Data represents percent vessel constriction in diameter ± SEM. *p<0.05 **p<0.01, and ***p<0.001.

Having established the importance of eNOS in arteriole remodeling and LN hypertrophy, as well as the pattern of arteriole remodeling throughout the course of infection, we considered initial reports that described increase in vessel diameter three days p.i., but also demonstrated a blunted response to inhibition to NOS [4]. Initial reports by our group noted that three days p.i., the feed arteriole demonstrated a blunted response to inhibition to NOS. We wondered if this blunted response continued throughout the course of infection similar to remodeling of the arteriole. We examined vessel response to L-NAME perfusion over the intravital microscopy preparation of the feed arteriole of C57 BL/6 mice zero, one, three, five, seven, nine, and thirty-five days p.i. (Figure 4A, B). Similar to previous findings the arteriole showed blunted constriction to L-NAME three days p.i., however, this blunted response was not noted one day p.i., but did persist through day five (Figure 4A). Notably, by day 7 response had returned to values similar seen at day zero and remained there through to day thirty-five (Figure 4B). This led us to conclude that like arteriole remodeling, the percentage of vessel constriction in response to NOS inhibition changes during the course of infection; however, the pattern of response to inhibition is different than vessel remodeling.

As arteriole remodeling was dependent on CD4^+^ T cells, we investigated whether the same mechanism was regulating NOS activity in the arteriole. To address this, the effect on L-NAME treatment on vessel diameter was studied in CD4^-/-^ mice zero, three, and seven days p.i. (Figure 4C) and showed that in the absence of CD4^+^ T cells, the arteriole still shows a blunted response to NOS inhibition at three days p.i., which returns to baseline levels by seven days p.i.. Notably, mice depleted of CD4^+^ T cells showed similar results to CD4^-/-^ mice (data not shown). Together, these data suggest that during infection the LN feed arteriole has decreased NOS activity contributing to vessel diameter three and five days p.i. and that this reduction, unlike the mechanism of remodeling, is not dependent upon CD4^+^ T cells. This, in turn, leads to the conclusion that the mechanism governing NOS activity within the LN feed arteriole during infection is different than that regulating vessel remodeling.

### Remodeling of the Lymph Node Feed Arteriole is TNFα dependent

**Figure 5:**
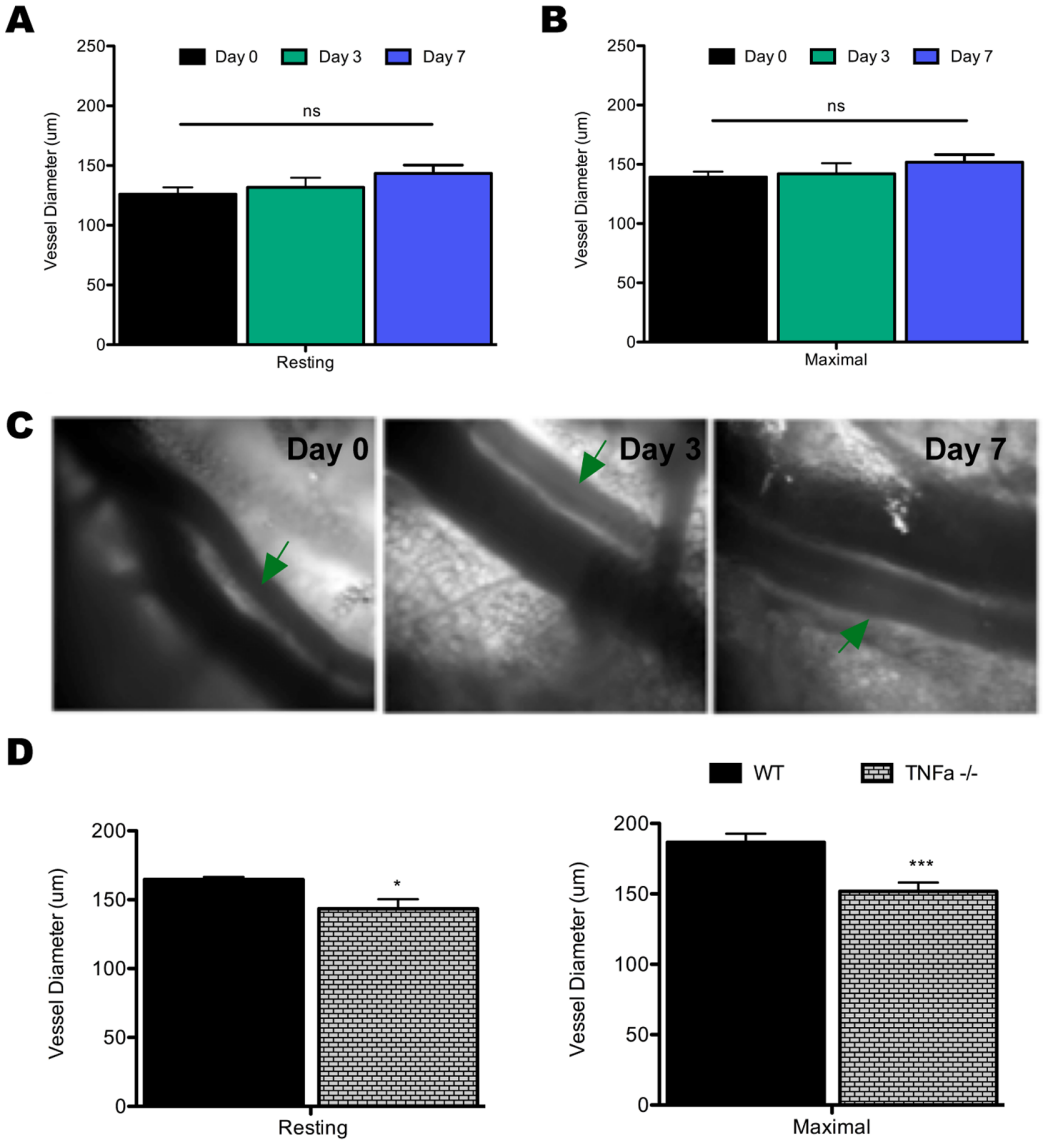
Resting arteriole diameter at days zero, three, and seven after i.vag. infection with TK^-^HSV-2 in TNFα^-/-^ mice (A). Resting vessel diameters were recorded after at least sixty minutes of equilibration with superfused physiological saline solution where vessel diameter, in which arterioles had consistent diameters for at least the last thirty minutes of equilibration. Maximal arteriole diameter (+SNP) at days zero, three, and seven (**B**). Data represents mean diameter (A,B) ± SEM wherein n =  5, 4, and 4 for days 0, 3, and 7, respectively. Representative images of resting diameter TNFα^-/-^ arterioles following infection at days zero and seven after infection (**C**). In each image the arteriole is indicated by green arrows. Relative comparison of resting and maximal diameter wild-type C57 BL/6 (WT) and TNFα^-/-^ mice at seven days post-infection (**D**). *p<0.05, **p<0.01, ***p<0.001, ns =  not significant.

Having established eNOS and CD4^+^ T cells as key mechanistic regulators of arteriole remodeling, LN hypertrophy and cellularity, and on-set of adaptive immunity, we sought to further elucidate the mechanism of arteriole remodeling. Based on previous studies demonstrating TNFα as a key regulator of LN hypertrophy and cellularity and as mediator of vascular changes [19]–[21], the ability of the LN feed arteriole to remodel during infection in the absence of TNFα was examined. *In vivo* measurement of the arteriole in TNFα deficient mice (Tnf^-/-^) shows that, in stark contrast to mice with intact TNFα signaling as previously reported [9], loss of TNFα results in failure to induce outward remodeling of the arteriole (Figure 5). This is evident in measurement of the arteriole at its resting equilibrated state (Figure 5A, C) or at induced maximal vessel diameter in comparison to day zero controls (Figure 5B).

Notably, TNFα deficient mice appear to have innately larger LN feed arteriole prior to infection than we have previously noted in wild-type C57 BL/6 background controls. However, we have previously noted this in other genetically manipulated strains and have always found that pharmacological recapitulation of the genetic knockout yields the same results as the knockout itself (data not shown). Additionally, when comparing LN arteriole diameters of TNFα deficient mice to wild-type mice at day seven p.i., when maximal arteriole remodeling should be observed, a significant decrease in both resting and maximal diameters is seen despite TNFα deficient mice having intrinsically larger arterioles prior to infection (Figure 5D).

**Figure 6:**
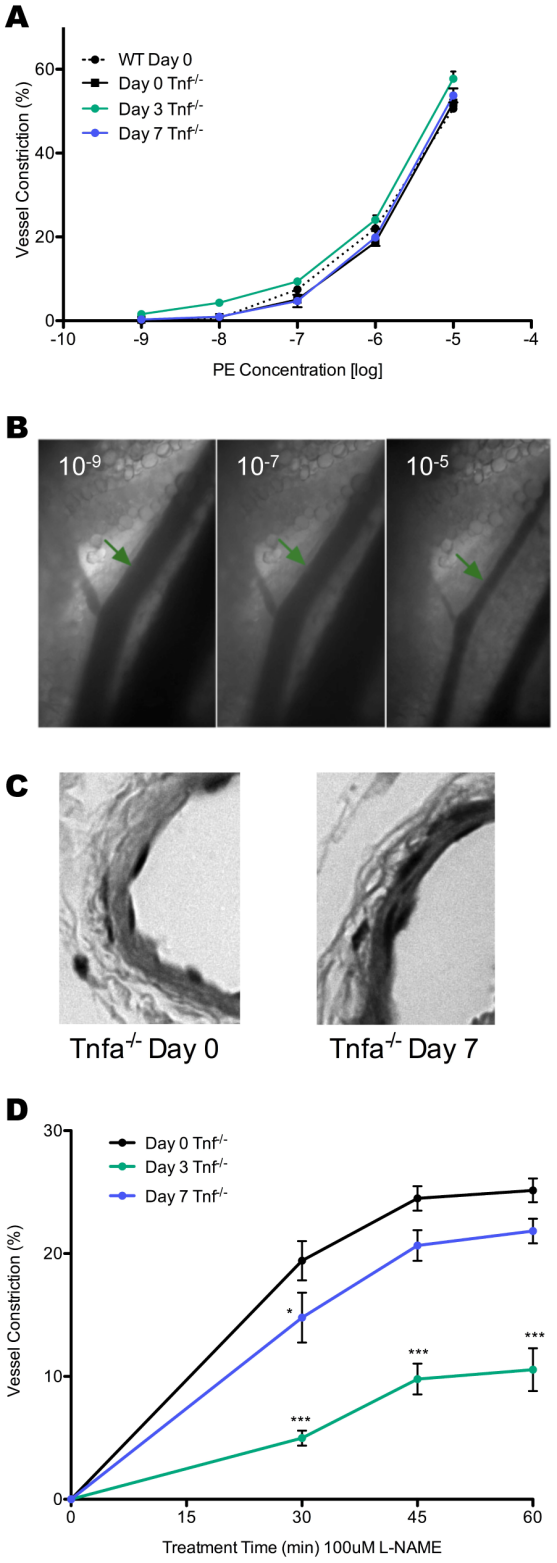
Phenylephrine (PE) dose response (percent vessel constriction) from 10^−9^M to 10^−5^M at zero (n = 5), three (n = 4), and seven (n = 4) days p.i. in TNFα^-/-^ mice in comparison to WT C57 BL/6 mice (n = 5) (A). Representative images of lymph node arterioles following at day zero p.i. prior to PE application and following superfusion of 10^−9^, 10^−7^, and 10^−5^M PE (B). In each image the arteriole is shown to the upper right of the LN venule and indicated by a green arrow (C). Representative images of H&E stained arteriole cross-sections in TNFα^-/-^ mice zero and seven days p.i. Percentage of vessel constriction in TNFα^-/-^ mice in response to perfusion of 100μM L-NAME at days zero (n = 5), three (n = 4), and seven (n = 4) following infection (D). Data represents mean vessel constriction (A, C) ± SEM where one arteriole was studied per animal. *p<0.05, **p<0.01, and ***p<0.001.

**Figure 7:**
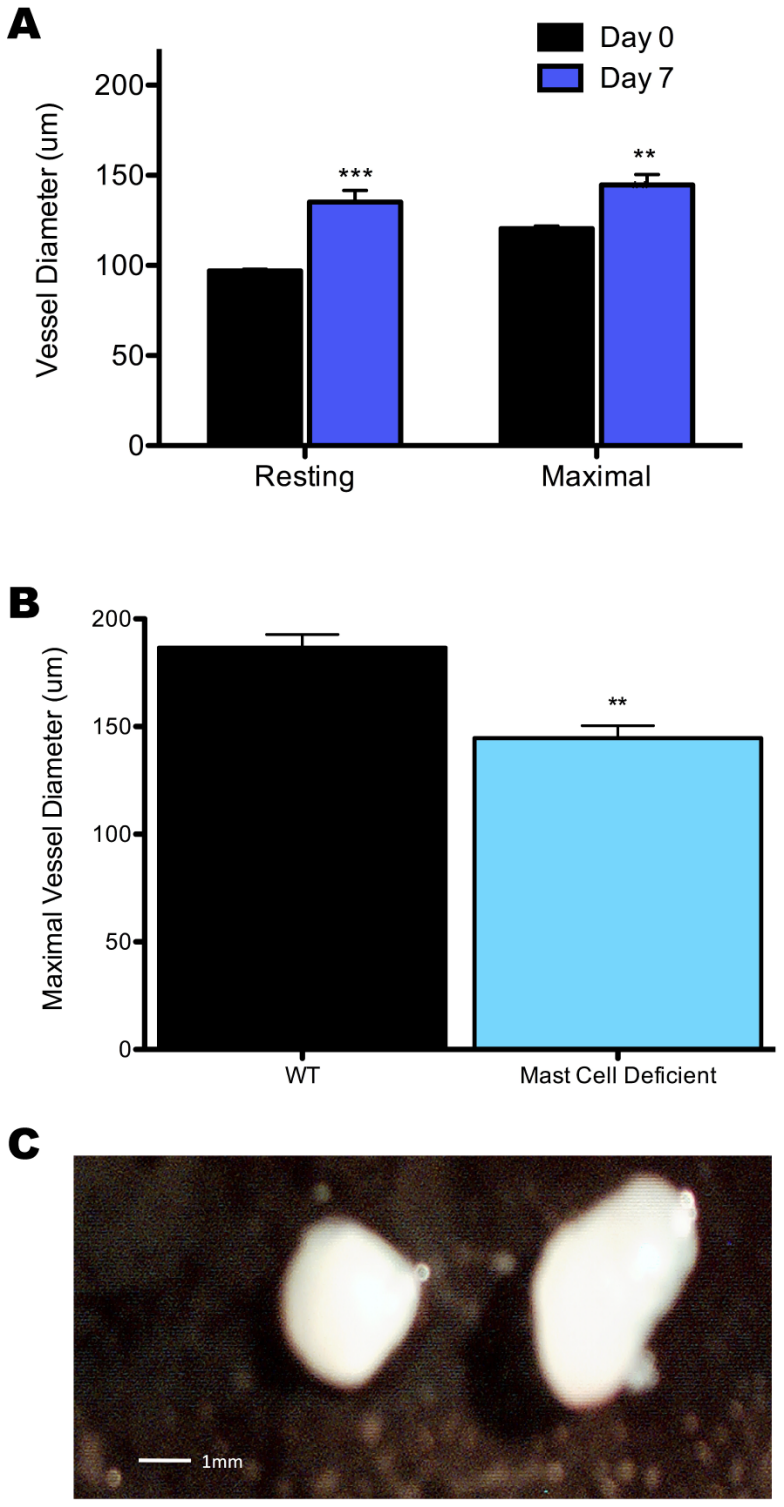
Resting and maximal (+30µM Nifedapine) arteriole diameter at zero (n = 4) and seven (n = 5) days after i.vag. infection in mast cell deficient mice (A). Comparison of maximal arteriole diameter in wild-type (C57 BL/6) (n = 5) and mast cell deficient mice (n = 5) at day seven following infection (B). Representative images of lymph nodes from mast cell deficient mice at days zero and seven following infection (C). Data represents mean vessel diameter (A, B) ± SEM where one arteriole was studied per animal. *p<0.05, **p<0.01, and ***p<0.001.

Although they demonstrated deficiency in arteriole remodeling, TNFα deficient arteriole still showed excellent response to PE that was comparable to wild-type day zero controls (Figure 6A,B). Additionally, H&E stained vessels showed no change in vessel morphology (Figure 6C) and loss of TNFα did not change vessel response to L-NAME at days three and seven during infection; arteriole response to application of L-NAME at day zero in TNFα deficient mice was similar to wild-type mice, and a significant decrease in response is seen at day three p.i., which returns to normal levels by day seven p.i. (Figure 7D). Collectively, this data demonstrates that TNFα is required for LN arteriole remodeling during infection, but does not mediate the transient drop in vessel response to NOS inhibition.

### Mast Cells Contribute to LN Arteriole Remodeling

Reports that cited TNFα as a mediator of LN expansion provided evidence that the key source of TNFα was mast cells; significant levels of mast cell degranulation is noted within two hours of challenge and mice given TNFα deficient mast cells have blunted LN hypertrophy [19], [20]. This, in combination with the requirement for TNFα demonstrated above, prompted investigation of the requirement for mast cells in arteriole remodeling. Mice deficient in mast cells showed significant remodeling seven days p.i., in comparison to day zero controls (Figure 7A). However, vessels were still significantly smaller than day seven wild-type mice (Figure 7B). Furthermore, mast cell deficient mice demonstrated only a moderate level of LN hypertrophy (Figure 7C). Collectively, this suggests that arteriole remodeling is partially dependent upon mast cells and that mast cells may contribute to the magnitude of arteriole remodeling

## Discussion

This study is the first characterization of LN feed arteriole remodeling throughout the complete course of infection. Our findings build on our pervious findings that remodeling is first notable one day p.i., and that expansion continues until at least day seven p.i. by demonstrating that remodeling, in terms of both resting and maximal arteriole diameter, peaks at day seven, after which it begins to return to a size similar to that seen before infection [4], [9]. This provides the complete characterization of the kinetics and magnitude of arterial remodeling, which is a vital step forward in our understanding of LN arteriole function and physiology; it allows us to begin to make connections between alternations within the feed arteriole and other events within the LN. The timeline of remodeling, being noted as early as one day p.i., and continuing until day seven p.i., corresponds to LN as cellular accumulation within the node has been shown to increase until at least day five p.i., as well as the time-course of CTL priming and migration back to the infection site [4], [22]. It also correlates with the timeline noted for other forms of LN vascularity; initial studies of increased LN blood flow also support vascular growth mimicking the kinetics of immune response; tracing of blood-borne microbeads indicate that blood flow to the LN rapidly increases following infection and remains elevated for at least five days, with increasing blood flow correlating to increased LN weight. Microangiography also noted increased vascularization within a day of immunization with a peak at day five, which subsequently returned to baseline level [23]. Total HEV surface area, quantified by antibody staining, was noted to increased following immunization [24], with Soderberg and colleges reporting an increase in HEV number proportional to increase in LN size until at least day four after infection [4]. Additionally, an increase in internal HEV diameter increasing rapidly after immunization to a peak of 3-times greater at day three, with subsequent decrease to baseline diameter by day twelve [25]. Collectively, this shows that the pattern of arteriole remodeling correlates with other significant changes in LN vascularity and processes inducing adaptive immune response and fills a significant gap in our understanding of LN physiology by identifying the LN arteriole as an important dynamic factor influencing LN function.

The finding of reversible outward eutrophic remodeling was also exciting, as although changes in vascularity have been shown to occur in other vascular beds during infection, in all cases this is pathogenic and irreversible. For example in chronic inflammation, which can be the precursor to many diseases such as airway disease, where arterioles, capillaries and venules are all pathogenically and chronically enlarged or in pulmonary arterial remodeling in response to hypertension [26], [27]. In contrast, remodeling of the LN arteriole is not pathogenic, as highlighted by the return of vessel diameter to baseline that coincides with the clearance of infection, and as such, may shed light on pathogenic forms of arteriole remodeling in the future. Notably, outward eutrophic remodeling would require increased EC size or number in order to maintain vessel wall width around a significantly larger lumen. The ability of the arteriole to adapt in this manner is supported by studies that show ECs numbers increase in the LN by day three and peak at day seven following stimulation in a DC, L-selectin, and VEGF dependent manner [14], all of which link closely with our previous findings of the role of CD4^+^ T cells and DCs, and our current finding of eNOS dependency. Furthermore, EC proliferation within stimulated LNs is present in both PNAd^+^ (eg. HEVs) and PNAd^-^ (eg. arteriole, lymphatics) cells within the LN, which supports the potential for ECs proliferation within the LN arteriole during remodeling. Notably, the kinetics of endothelial cell division and vascular changes in non-LN settings also correspond to the current findings within the LN feed arteriole. For example, in the context of infection leading to inflammatory airway disease, increased arteriole diameters peaked seven days following infection and was accompanied by increased EC proliferation indicated by BrdU incorporation which peaked five days after infection and stopped at day nine [26], [27]. Similar patterns are also seen in angiogenesis where EC proliferation has been shown to peak around day three and persist through day six in the context of both graft-versus host and hypersensitivity type immune responses [28], [29]. Beyond endothelial cell proliferation, EC hypertrophy as also been noted in HEVs following stimulation, which subsequently returned to baseline and is also be an indicator or rapid EC proliferation [25], [26]. Therefore, a general conclusion that the dynamics of EC proliferation and hypertrophy, as noted within the LN and in other non-LN environments, corresponds to the timeline of arteriole remodeling within the LN can be drawn. This is an important conclusion as outward eutrophic remodeling, as is observed in the LN arteriole, would require increased EC size or number, and as such, supports the future hypothesis that LN arteriole remodeling is driven by EC proliferation, hypertrophy and/or recruitment and ultimately supports and further investigation of EC function in the LN arteriole in the future.

Beyond being the first complete characterization of LN arteriole remodeling, these are also the first studies to implicate eNOS and NO in the regulation of LN vascular expansion and hypertrophy. The role of eNOS and NO in the vasculature of the LN and its subsequent impact on immune response has never been studied outside of our initial report [4]. Here we demonstrated, both genetically and pharmacologically, that the mechanism of arteriole remodeling is eNOS dependent and that constriction as a result of L-NAME perfusion over the arteriole is decreased three and five days p.i.. Blunted response to NOS inhibition three and five days p.i., implies a change in NOS activity and subsequently NO production on these days. Alternatively, it could imply a change in NO bioavailability and NO acting upon the vessel and influencing diameter. Interestingly, this process was not dependent on CD4^+^ T cells as arteriole remodeling was noted to be. While the mechanism of the blunted response to NOS inhibition remains to be elucidated, so does its function. One hypothesis about potential function is to facilitate migration into the lymph node, which is supported by the lack of hypertrophy seen in eNOS^-/-^ LNs following infection. Additionally, previous studies have shown NO to inhibit T cell rolling along the endothelium, with inhibition of NOS leading to increased rolling, adhesion, and emigration [30]–[32], therefore, the arteriole may facilitate T cell rolling by down-regulating NO production. However, complete shut-down of NO would be detrimental, as evidenced by the lack of LN hypertrophy in eNOS^-/-^ mice and with pharmacological inhibition of NOS with L-NAME treatment. Lack of eNOS may be inhibitory to LN arteriole remodeling due to its impact on VEGF, which is key in LN vascular growth and EC proliferation [14]. A requirement for eNOS has been shown previously to be required for a response to VEGF outside the LN, with loss of eNOS results in reduction in angiogenesis, arteriogenesis and mural cell recruitment [18], [33] and exogenous delivery of eNOS has been shown to increase VEGF expression and give rise to mature phenotype of newly formed arterioles and capillaries [15]. Alternatively, change in shear stress triggering EC proliferation in response to high flow, which would be required for increased delivery of blood to the LN and outward eutrophic remodeling, may also be a mechanism behind the requirement of eNOS dependency in the LN [34], and future studies will focus further investigation of such mechanism.

It is also arguable that the blunted response to L-NAME, suggesting decreased NO, is contradictory to increases in VEGF that was previously proposed to be a player in arteriole remodeling and is key in LN ECs proliferation during infection [14]. This is because VEGF is known to activate eNOS. Following stimulation of VEGF receptors, increased eNOS expression results from activation of the PI3K/Akt pathways leading to release of eNOS from caveolin-1, which binds eNOS and decreases its activity, with subsequent increased eNOS activity due to phosphorylation[35], [36]. This means that decreased eNOS would be associated with decreased VEGF, both of which are contradictory to vascular growth. However, a theory of two separate pathways may harmonize these opposing points. The pathway first being that VEGF levels are unregulated in LN to facilitate vascular growth and that VEGF is secreted and/or signaling is directed upstream to the HEVs and subsequently the arteriole. The second that decreased NO induced vasodilation at days three and five p.i. are specific to the arteriole and are not affect by VEGF levels in the LN, but may play a role in supporting VEGF secretion by increasing lymphocyte trafficking. Notably, these hypotheses require future investigation. However, even before such future studies begin, the work presented here represent a critical step forward in understanding LN physiology and vascularity, arteriole remodeling, and interaction between the microvasculature, NO, and immune response, and provide the means on which to begin to fully elucidate the therapeutic potential of regulating adaptive immune response onset in the LN via vascular manipulation.

The second part of our investigation of vascular mediators of LN arteriole expansion focused on TNFα, and demonstrated the process to be TNFα dependent. TNFα is a known regulator of vascular remodeling, EC proliferation, and T cell migration but is known to act early in the process of vascular remodeling. This suggests that TNFα may act early in the LN arteriole remodeling process to induce initial stages of EC proliferation and T cell migration. Notably, in the case of M. *pulmonis* infection, increase in TNFα preceded vascular remodeling events and blockade of TNFα resulted in decreased VEGF levels, and leukocyte trafficking and slowed the process of remodeling of blood vessels [21], suggesting that TNFα in the LN may be required for increased VEGF. TNFα may also be facilitating VEGF expression and subsequent vascular expansion by facilitating DC migration into the LN [21], [37], [38]. Later in the arteriole remodeling and infectious process, TNFα may also be acting to limit the magnitude of the inflammatory response within the LN as TNFα been shown to up regulate the A_2B_ receptor, a receptor for CD73-generated adenosine known to be expressed on HEVs, and the absence of CD73 results in increased trafficking into the LN and vascular permeability, which is abrogated by pharmacological blockade of the A_2B_ receptor. These findings suggest that CD73-generated adenosine may act to limit inflammatory associated permeability within the LN, facilitated by TNFα regulation of the A_2B_ receptor [39].

Finally, our studies on the potential requirement of mast cells for arteriole remodeling showed a partial requirement for mast cells; mice deficient in mast cells showed a decreased magnitude of remodeling. However, more experiments will be required to definitively highlight the role of mast cells in remodeling, as there are more robust mast cell deficient mouse models are available. Previously, McLachlan and colleges [19], [20] showed that TNFα released from mast cells was transmitted upstream to the LN and facilitated LN hypertrophy, including DC and lymphocyte accumulation. This would suggest that if arteriole remodeling was observed to be dependent on lymphocyte accumulation and interaction with DCs and TNFα, it should also be dependent on mast cells. However, this cellular accumulation was transient in studies by McLachlan and colleges happened early, spiking at twenty-four hours and declining by forty-eight hours, indicating requirement for TNFα derived from mast cells acts early in the remodeling process, and that TNFα derived from an alternative sources may act throughout the remainder of the remodeling process, although mast cell derived TNFα may facilitate increased magnitude of response, subsequently suggesting that this may be used in the future to increase the magnitude of arteriole remodeling and, therefore, the rate of screening for cognate lymphocytes and the induction of adaptive immune response.

## Materials and Methods

### Viruses, and Reagents

Depo-Provera (5 mg/mouse) treated female mice were intravaginally (ivag) infected with 10^6^ pfu of thymidine kinase mutant HSV-2 strain 186TKΔKpn or with uninfected Vero cell lysate (mock infected) as described previously [4]. All other reagents were from Fisher Scientific, unless specified otherwise.

### Mice

Female C57 BL/6 (WT, stock ^#^000664), CD4^+^ T cell deficient mice (CD4^-/-^, B6.129S2-CD4<tm1Mak>/J, stock ^#^002663), mast cell deficient mice (compound heterozygous WBB6F1/J-Kit<W>/Kit<W-v>/J, stock ^#^100410), tumor necrosis factor alpha deficient mice (Tnf ^-/-^, homozygous B6.129S-Tnf<tm1GK1>/J, stock ^#^005540) and eNOS^-/-^ (homozygous B6.129P2-NOS3<tm1Unc>/J, stock ^#^002684) mice, >8 wks old, were purchased from The Jackson Laboratory. Additionally, homozygous eNOS^-/-^ breeder pairs were also purchased from The Jackson Laboratory and bred in the Northern Health Sciences Research Facility (NHSRF) at UNBC; all procedures were approved by the University of Northern BC Animal Care and Use Committee.

### L-NAME Treatment

Female C57 BL/6 mice were treated with Nitro-L-arginine methyl ester hydrochloride (L-NAME, Sigma-Aldrich, catalog ^#^N5751). L-NAME was dissolved in drinking water at a concentration of 0.5 mg/ml changed daily. This method has been shown to be effective at blocking NOS expression in the context of vascular remodeling and lymphocyte accumulation in asthma models in the lung [40]. Treatment was administered for fourteen days prior to initial treatment with Depo-Provera and continued throughout the course of Depo-Provera treatment and infection.

### Intravital Microscopy

Mice were anesthetized with sodium pentobarbital (90 mg/kg). Surgical exposure of the LN feed arteriole was modeled after the model described by von Andrian [5] and previously described [9], [10]; midline incision was made along the ventral surface of the abdominal cavity. The skin was retracted and pinned onto a pedestal of transparent Sylgard 184 (Dow Corning) while continuously superfused with a bicarbonate-buffered physiological salt solution (PSS) equilibrated with 5% CO_2_/95% N_2_ (pH 7.4, 34°C). This allowed for observation of the inguinal LN. Surrounding adipose tissue was cleared and the main arterial segment lying adjacent to the LN was visualized and evaluated. The preparation was secured on an intravital microscope and the feed arteriole was observed at a total magnification of 950× using video microscopy. Internal vessel diameter was determined from the width of the red blood cell column using a video caliper. The same vessel segment was studied in each mouse per group. The resting internal diameter was measured following 60 min equilibration with PSS. The effect of a smooth muscle- (phenylephrine, PE) specific agonist (Sigma-Aldrich) was evaluated by cumulative addition (1 nM to 10 µM) to the superfusate. At each concentration, the stable arteriolar diameter was recorded before the next increment. The preparation was then superfused with control PSS to recover (∼35 min) and the other agonist was evaluated. Then the preparation was equilibrated with *N*w-nitro-L-arginine (100 µM for 60 min) to inhibit NOS. The preparation was then equilibrated with sodium nitroprusside (SNP, 100 µM, Sigma-Aldrich) to obtain maximal diameter *in vivo*. In the case of eNOS^-/-^ mice, maximal vessel diameter was induced through preparation equilibration with nifedipine (30 µM, Sigma-Aldrich).

### Tissue Collection and Staining

LN feed arteriole segments were isolated from intravital preparations prior to sacrifice; Preparations were superfused with PSS containing 100 µM SNP and the feed arteriole was subsequently isolated and fixed in 10% buffered formalin. Vessels processing, staining, and IHC were preformed by Wax-it Histology Services Inc. Lymph nodes were harvested following euthanasia and stored in 10% buffered formalin.

### Statistical Analyses

All statistical analyses were performed with GraphPad Prism 5.0 (GraphPad Software, San Diego California USA, www.graphpad.com). Data was analyzed using one-way and two-way repeated-measures analysis of variance with Bonferroni and Dunnett's Multiple Comparison post tests, and paired T-tests with statistical significance defined as *p<0.05, **p<0.01, and ***p<0.001.
